# The role of intrinsic motivations in attention allocation and shifting

**DOI:** 10.3389/fpsyg.2014.00273

**Published:** 2014-04-01

**Authors:** Dario Di Nocera, Alberto Finzi, Silvia Rossi, Mariacarla Staffa

**Affiliations:** ^1^Dipartimento di Matematica e Applicazioni, Università degli Studi di Napoli “Federico II,”Napoli, Italy; ^2^Dipartimento di Ingegneria Elettrica e Tecnologie dell'Informazione, Università degli Studi di Napoli “Federico II,”Napoli, Italy

**Keywords:** attention shifting, curiosity, intrinsic motivations, reinforcement learning, action selection

## Abstract

The concepts of attention and intrinsic motivations are of great interest within adaptive robotic systems, and can be exploited in order to guide, activate, and coordinate multiple concurrent behaviors. Attention allocation strategies represent key capabilities of human beings, which are strictly connected with action selection and execution mechanisms, while intrinsic motivations directly affect the allocation of attentional resources. In this paper we propose a model of Reinforcement Learning (RL), where both these capabilities are involved. RL is deployed to learn how to allocate attentional resources in a behavior-based robotic system, while action selection is obtained as a side effect of the resulting motivated attentional behaviors. Moreover, the influence of intrinsic motivations in attention orientation is obtained by introducing rewards associated with curiosity drives. In this way, the learning process is affected not only by goal-specific rewards, but also by intrinsic motivations.

## Introduction

*Attention* and *intrinsic motivations* play a crucial role in cognitive control (Posner et al., 1980) and are of great interest in cognitive robotics. Indeed, attentional mechanisms and motivational drives are strictly involved in the process of guiding and orchestrating multiple concurrent behaviors. Attentional mechanisms, beyond their role in perception orientation, are also considered as key mechanisms in action selection and coordination (Posner et al., 1980; Norman and Shallice, 1986). The capability of selecting and filtering the information is associated with the process of focusing cognitive and executive resources toward the stimuli that are relevant for the environmental and behavioral context. On the other hand, another key factor affecting action selection is represented by the so called intrinsic motivations such as the curiosity (Baldassarre, 2011), which can indirectly affect action selection because of its influence on attentional shifting. For instance, the curiosity drive can attract the attentional focus toward novel stimuli and, consequently, can elicit the execution of actions which are not directly related to the current behavior or goal. Albeit there is not a clear consensus on how intrinsic motivations differ from the extrinsic ones (Baldassarre, 2011), their role in pushing human/animal beings to spontaneously explore their environment (Baldassarre and Mirolli, 2013) and to execute this activity only for their inherent satisfaction (Ryan and Deci, 2000), rather than for satisfying some basic needs such as hunger or thirst (White, 1959; Berlyne, 1960), is widely accepted.

In this work, we focus on the intrinsic motivation provided by the curiosity, which is considered as the main drive for humans to explore novel situations and to learn complex behaviors from experience (Berlyne, 1954; Litman, 2005). Recent studies have also shown that both attention and curiosity are strictly related to the dopaminergic system responsible for action driving. It is widely accepted, indeed, that dopamine affects both the reward excitement, fundamental in the learning process, and the demand of more attention by novel stimuli (Nieoullon, 2002; Redgrave and Gurney, 2006; Jepma et al., 2012). Unpredicted events can generate intrinsic reinforcement signals, which support the acquisition of novel actions. In particular, it has been shown that the dopamine release is triggered not only in response to unexpected environmental changes and goal-directed action-outcome learning (Heidbreder and Groenewegen, 2003; Dalley et al., 2004), but also in response to the detection of novel events (Lisman and Grace, 2005).

The typical approach adopted for modeling the dopamine-like rewarding system (Montague et al., 1996) and for coping with the problem of treating intrinsic motivations (Barto et al., 2004; Mirolli and Baldassarre, 2013) is represented by the well known Reinforcement Learning (RL) process. Recent works have been proposed to incorporate models for novelty (Marsland et al., 2000) and curiosity (Schmidhuber, 1991) within Motivated RL algorithms (Barto et al., 2004) providing accounts for behavior adaptation, action selection learning, mental development, and learning of hierarchical collections of skills depending on the robot experience (Kaplan and Oudeyer, 2003; Barto et al., 2004; Oudeyer et al., 2007; Schembri et al., 2007; Singh et al., 2010; Baranes and Oudeyer, 2013). Typically, within these approaches, RL is used to directly model and generate the action selection strategies. In contrast, we propose a system where RL is deployed to learn attentional allocation and shifting strategies, while action selection emerges from the regulation of attentional monitoring mechanisms (Di Nocera et al., 2012), which can be affected by the intrinsic motivation of curiosity. Our curiosity model is inspired by the interest/deprivation model proposed by Litman (2005), which captures both optimal-arousal and curiosity-driven approaches of curiosity modeling. Following this approach, attentional shifting mechanisms can be generated taking into account not only extrinsic motivations, like mission goals and primary needs satiation, but also intrinsic motivations, like the need of acquiring knowledge (Litman's deprivation model) and the attraction toward novel stimuli and opportunity of learning (Litman's interest-based model). In this context, we aim to investigate whether our account of curiosity and attentional regulation learning is feasible and effective for the generation of attentional allocation and shifting strategies, whose side effect is an adaptive emergent behavior for the robot. We are also interested in the impact of our model of curiosity on the learning process. Specifically, we want to assess whether the proposed intrinsically motivated system affects the progress in learning.

We detail the approach by describing our intrinsically motivated RL model and analyzing its performance in a simulated survival domain. In this scenario, the robot is engaged in survival tasks such as finding food or water, while avoiding dangerous situations. The goal is to learn attentional allocation and shifting policies that allow the robot to survive in the particular environment. The system evaluation is based on a comparison between the performance of the attentional policies, which are learned with the curiosity model, with respect to the ones generated without taking into account the curiosity drive. The collected results show that our intrinsically motivated learning approach is feasible and effective. Indeed, the curiosity-driven learning system allows us to find satisfactory attentional allocation and shifting policies showing a faster convergence of the learning process, safer policies of the selected action, and a higher wellness state of the robotic system in terms of energy gained during the exploration of the environment. In particular, in the curious setting the robot behavior seems more flexible because endowed with an additional capacity of adaptation. Indeed, different attentional allocation (or shifting) policies, and consequently, various action selection policies, can be defined depending on the current level of curiosity.

## Material and methods

### Attentional shifting system

In this work, we refer to the attentional framework introduced by Burattini et al. (2010). Here, the attentional system is modeled as a reactive behavior-based system (Brooks, 1986; Arkin, 1998), endowed with internal attentional mechanisms capable of distributing and shifting the attention among different concurrent behaviors depending on the current saliency of tasks and stimuli. These attentional mechanisms allow the robotic system to supervise multiple concurrent behaviors and to efficiently manage limited resources. In contrast with typical works on visual attention (Itti and Koch, 2001), the Burattini et al. (2010) approach is not concerned with the orientation of the attention in the space (i.e., the field of view), but it is about the executive attention (Posner et al., 1980) and the temporal distribution of the attentional resources needed to monitor and control multiple processes. This model of attention is inspired by Pashler and Johnston (1998), where the attentional load due to the accomplishment of a particular task is defined as the quantity of attentional time units devoted to that particular task, and by Senders (1964), where attentional allocation and shifting mechanisms are related to the sampling rate needed to monitor multiple parallel processes. In particular, Burattini et al. (2010) propose a frequency-based model of attention allocation, where the increment of the attention due to salient stimuli is associated with an increment of sensors sampling rate and of the behavior activations. Specifically, starting from a behavior-based architecture, each behavior is endowed with an internal clock regulating its activation frequency and sensory sampling rate: the higher the sampling rate, the higher the resolution at which the behavior is monitored and controlled. The internal clock can increase or decrease the attentional state of each behavior with respect to salient internal/external stimuli by means of suitable attentional monitoring functions. In this context, the internal stimuli are modeled as internal needs, such as, for example, thirst or hunger, while the external stimuli are associated with salient events or discontinuities perceived in the external environment.

**Figure 1:**
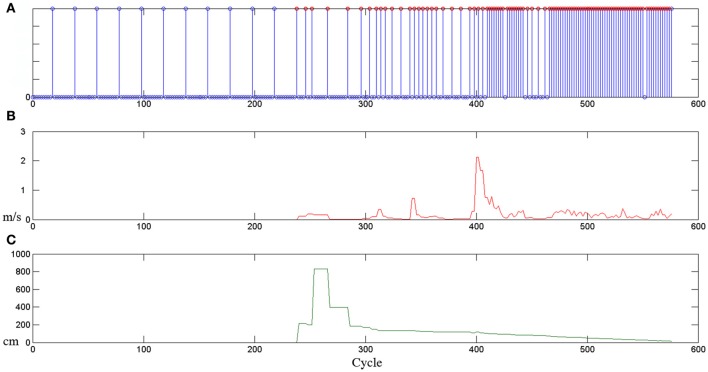
**Example of the attentional allocation strategies presented in Sidobre et al. (2012). (A)** The sampling rate associated with the behavior of *giving an object* changes depending on internal or external stimuli: **(B)** the human hand speed and **(C)** the distance between the human hand and the robot end-effector.

An explicative example of this behavior-based attentional system at work is presented in Figure 1. The plot shows how the sampling rate of a behavior (for example a *give* task) changes (see Figure 1A) depending on different stimuli (for example, the human hand speed, in Figure 1B, and the distance between the human hand and the robot end effector, in Figure 1C). It is possible to observe that if non-salient stimuli are presented to the behavior, the attentional process monitors the environment in a relaxed manner, instead, if something salient happens, the clock frequency of the behavior is enhanced and more attention is consequently paid toward the stimulus. This general model permits to monitor and control different internal and external processes, shifting, from time to time, the allocation of computational and operational resources. Notice that, this adaptive frequency implicitly provides a mechanism for behaviors prioritization. Indeed, high-frequency behaviors are associated with activities with a high relevance and priority in the current operational context.

#### Formalization of the model

Following the approach of Burattini and Rossi (2008), we consider a Behavior-based architecture (Brooks, 1986; Arkin, 1998), where each behavior is endowed with an attentional mechanism represented by an internal adaptive clock.

**Figure 2:**
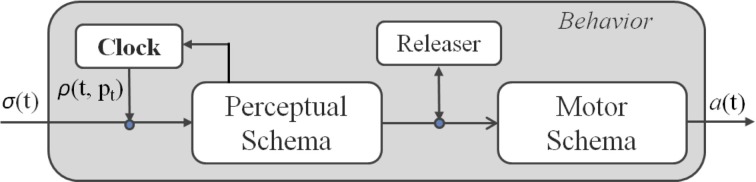
**Schema Theory representation of an attentional behavior**.

A schema theory representation (Arbib, 1998) of an attentional behavior is illustrated in Figure 2. This is characterized by a Perceptual Schema (PS), which elaborates sensor data, a Motor Schema (MS), producing the pattern of motor actions, and an attentional control mechanism, called Adaptive Innate Releasing Mechanism (AIRM), based on a combination of a clock and a releaser. The releasing mechanism works as a trigger for the MS activation (e.g., the view of a predator releases the escape behavior), while the clock regulates the sensors sampling rate and, consequently, the activation rate of the behaviors. The clock activation rate changes following an attentional monitoring strategy, which can adaptively increase or decrease the clock frequency, according to salient internal and external stimuli. More formally, the attentional mechanism is characterized by:

- An activation period *p*^*b*^ ranging in an interval [*p^b^_min_*, *p^b^_max_*], where *b* is the behavior identifier.
- A *monitoring function f*(σ^*b*^(*t*), *p*^*b*^_*t* − 1_): ℝ^*n*^→ ℝ that adjusts the current clock period *p^b^_t_*, according to the internal state of the behavior and to the environmental changes.
- A trigger function ρ(*t*, *p^b^_t_*), assuming a 0/1 value, which enables/disables the data flow σ^*b*^(*t*) from sensors to PS at each *p^b^_t_* time unit.
- Finally, a normalization function ϕ(*f*(σ^*b*^(*t*), *p*^*b*^_*t* − 1_)):ℝ → ℝ that maps the values returned by *f* into the allowed range [*p^b^_min_*, *p^b^_max_*].

The clock period at time *t* is regulated as follows:

(1) $$p_{t}^{b}=\rho \left(t,p_{t\;-\;1}^{b}\right)\cdot \phi \left(f(\sigma^{b}(t),p_{t\;-\;1}^{b}\right)+\left(1-\rho (t,p_{t\;-\;1}^{b})\right)\cdot p_{t\;-\;1}^{b}$$

That is, if the behavior is disabled, the clock period remains unchanged, i.e., *p^b^*_*t* − 1_. Otherwise, when the trigger function is 1, the behavior is activated and the clock period changes according to the ϕ(*f*). In order to learn attentional monitoring strategies, various methods such as Differential Evolution (Burattini et al., 2010) and RL techniques (Di Nocera et al., 2012) have been deployed, respectively for off-line and on-line tuning of the parameters regulating the attentional monitoring functions. In the following sections, we will present an intrinsically motivated RL (IMRL) approach to the attentional allocation problem in our frequency-based model of attention.

### Intrinsic motivations: curiosity model

Curiosity is an appetitive state involving the recognition, pursuit, and intense desire to investigate novel information and experiences that demand one's attention. In literature, we find two main theoretical accounts of curiosity: the *optimal arousal model* and *curiosity-drive* theory. The curiosity-drive model assumes that the main drive of curiosity is the reduction of uncertainty: novel and ambiguous stimuli cause a need for coherence restore that reduces the uncertainty. This reduction is considered as rewarding. This model is supported by studies showing that unusual situations are associated with approaching behaviors and attentional states (e.g., see the Loewenstein, 1994 knowledge gap/approach gradient). However, the curiosity-driven model cannot explain why biological organisms initiate exploratory behaviors without any stimuli. These situations are instead well explained by the optimal-arousal model (e.g., see the Spielberger and Starr, 1994 model). Following this model, the biological systems are associated with an homeostatic regulation of their arousal level: when the arousal level is under-stimulated, the organism is motivated to increase the arousal and to look for novel situations; in contrast, when the organisms is over-stimulated additional stimuli are evaluated as negative and associated with an avoidance behavior. While in the curiosity-drive model the reward is associated with uncertainty reduction, in the optimal arousal model, the induction of curiosity is directly rewarding. Also this model is not completely satisfactory, indeed, in this case an optimal arousal state should be maintained and the reward is directly associated with a feeling of interest, hence the gain of new knowledge could reduce this feeling and could be considered counter-productive (see Litman, 2005 for a discussion). A combination of these two approaches is proposed by Litman (2005) with the *interest/deprivation* model of curiosity. Here, both the satiation and the activation of curiosity can be rewarding: the interest-based curiosity is driven by novel stimuli and opportunity of learning, whereas the deprivation-based curiosity is driven by the uncertainty and the lack of knowledge. In Litman (2005) the interest/deprivation model of curiosity is then related to the neuroscience of the wanting and liking systems, which are hypothesized to underlie motivation and affective experience for a wide class of appetites (Berridge, 2003). In the Litman model, wanting is associated with deprivation and need of knowledge, while liking is associated with the expected pleasure due to learning and knowledge acquisition. In Table 1 we show the Litman's classification. Here, in the case of high level of wanting and liking, curiosity is due to a need of knowledge and it is sustained by an interest; if wanting is low, but liking is high, information seeking is motivated by pure interest; in contrast, if wanting is high and liking is low, the need of knowledge is not associated with the anticipation of a pleasure. Finally, when wanting and liking are low, also the curiosity drive is inhibited. In this paper, we exploit a model of curiosity that is inspired by the Litman (2005) interpretation of wanting and liking.

**Table 1:** **Litman's classification of curiosity states with respect to high and low levels of liking and wanting (Litman, 2005)**.

**Liking**	**Wanting**	
	**Low**	**High**
*Low*	LL: Ambivalent disinterest	LH: Need for uncertainty clarification
*High*	HL: Curiosity as a feeling of “interest”	HH: Curiosity as a feeling of “deprivation”

### Reinforcement learning for attentional shifting

Following the approach by Di Nocera et al. (2012), in this paper we exploit a RL algorithm to learn the attention allocation strategies introduced in section 2.1. In Di Nocera et al. (2012), a Q-learning algorithm is used to tune and adapt the frequencies of sensors sampling, while action selection is obtained as a side effect of this attentional regulation. In the following, we first recall the Q-learning algorithm and then we detail its application to the attentional shifting problem.

#### General description of the Q-learning algorithm

Q-learning (QL) (Watkins and Dayan, 1992) is a learning algorithm for a Markov Decison Process (MDP). A MDP is defined by a tuple (*S*, *A*, *R*, *P_a_*) where *S* is the set of states, *A* is the set of actions, *R* is the reward function *R* : *S*× *A*→ ℝ, with *R*(*s*, *a*) the immediate reward in *s* ∈ *S* after the execution of *a* ∈ *A*; *P*_*a*_ is the transition function *P_a_*: *S*× *A*× *S*→[0,1] ∈ ℝ, with *P*_*a*_(*s*, *a*,*s*') probability of *s*' ∈ *S* after the execution of *a* ∈ *A* in *s* ∈ *S*. A solution of a MDP is a policy π: S → A that maps states into actions. The *value function V*^π^ (*s*) is the cumulative expected reward from *s* ∈ S following π. The *q-value Q*(*s*, *a*) is the expected discounted sum of future payoffs obtained by executing the action *a* from the state *s* and following an optimal policy π^*^, i.e., *Q*(*s*, *a*) = {*R*_*t* + 1_ + γ V^*^(*s*_*t* + 1_) | *s*_*t*_ = *s*, *a_t_* = *a*}, with V^*^ associated to π^*^.

In QL techniques, the Q-values are estimated through the agent experience after being initialized to arbitrary numbers. For each execution of an action *a_t_* leading from the state *s_t_* to the state *s*_*t* + 1_, the agent receives a reward *r*_*t* + 1_, and the Q-value is updated as follows:

(2) $$\begin{array}{l} Q(s_{t},a_{t})\leftarrow (1-\alpha_{t})\cdot Q(s_{t},a_{t})+\alpha_{t}(R_{t\;+\;1}\\ \;+\gamma \cdot max_{a_{t+1}\in A}Q(s_{t\;+\;1},a_{t\;+\;1})) \end{array}$$

where γ is the discount factor (which determines the importance of future rewards) and α is the learning rate.

Different exploration policies can be introduced to select the action to be executed trying to balance exploration and exploitation. Analogously to Di Nocera et al. (2012), in this paper we consider a *Softmax* method that selects the action to be executed through a Boltzmann distribution (Sutton and Barto, 1998):

(3) $$P_{a}(a|s,Q)=\frac{\exp^{\frac{Q(s,a)}{\tau }}}{\sum_{b\;\epsilon A(s)}\exp^{\frac{Q(s,b)}{\tau }}}$$

Here, the temperature τ controls the exploration strategy: the higher the temperature, the closer the strategy is to a random policy (exploration); the lower the temperature, the closer the strategy is to *Q*(*s*, *a*) maximization (exploitation). Under suitable conditions (see, for example, Watkins and Dayan, 1992), this algorithm converges to the correct Q-values with probability 1 assuming that every action is executed in every state infinitely many times and α is decayed appropriately.

#### Q-learning for attentional regulation

In our setting, the QL algorithm is to be exploited to generate the attention allocation strategy. For this purpose, we introduce a suitable space state *S*^*b*^ for each attentional behavior, while the action space *A*^*b*^ represents a set of possible regulations of the clocks. Specifically, the action space spans a discretized set of possible allowed periods *P*^*b*^ = {*p^b^*_1_, …, *p*^*b*^_*k*_} for each behavior *b* (i.e., *A*^*b*^ coincides with *P*^*b*^). Since the current state *s*^*b*^ ∈ *S*^*b*^ should track both the attentional state (clock period) and the perceptive state, this can be represented by a pair *s*^*b*^ = (*p*^*b*^,σ^*b*^), where *p*^*b*^ ∈ P^*b*^ is the current clock period and σ^*b*^ ∈ X^*b*^ is for the current perceptive status. In particular, we consider the perceptive state of each behavior as a discretization of the behavior perceptive domain using *n* equidimensional intervals *X*^*b*^ = {σ^*b*^_1_, …, σ^*b*^_*n*_}. Therefore, the attentional allocation policy π^*b*^ : *S*^*b*^ → *A*^*b*^ represents a mapping between the current state *s*^*b*^ and the next attentional period *p*^*b*^ that should be learned by means of the QL algorithm. That is, given a reward function *R* for each behavior, the algorithm is to find the optimal attention allocation policy π^*b*^, i.e., for each state *s*^*b*^ ∈ *S*^*b*^, the activation period *p*^*b*^ ∈ P^*b*^ that maximizes the expected reward of that behavior.

The resulting Q-table for a generic attentional behavior in Di Nocera et al. (2012) can be described by the Table 2.

**Table 2:** **Q-values for a generic behavior, where *S*^*b*^ represents the state space**.

*S*^*b*^		*A*^*b*^			
		*p_1_*	*p_2_*	…	*p_k_*
σ_*1*_	*p*_1_	*Q*_11,1_	*Q*_11,2_	…	*Q*_11,*k*_
	…	…	…	…	…
	*p_k_*	*Q*_1*k*,1_	*Q*_1*k*,2_	…	*Q*_1*k,k*_
…	…	…	…	…	…
σ_*n*_	*p*_1_	*Q*_*n*1,1_	*Q*_*n*1,2_	…	*Q*_*n*1,*k*_
	…	…	…	…	…
	*p*_*k*_	*Q*_*nk*,1_	*Q*_*nk*,2_	…	*Q*_*nk,k*_

This approach to adaptive attentional allocation and action selection has been tested in a robotic setting (Di Nocera et al., 2012). Starting from this model we will design our model of *Intrinsically Motivated Reinforcement Learning*.

## Motivated RL for attentional shifting

In this section, we extend the RL approach to attention allocation presented above introducing the effects of the intrinsic motivation of curiosity. In particular, we rely on a curiosity model which is inspired by the interest/deprivation model proposed by Litman (2005) and adapted to the behavior-based setting we consider in this work. More specifically, analogously to Litman, we associated the liking mechanism to a direct reward related to novelty, however, our interpretation of the wanting system is slightly different. Indeed, in the place of the cognitive deprivation model introduced by Litman, which cannot be easily accounted within the simple behaviors we are concerned with, we relate the wanting mechanism to the need to explore and act. This is represented by a value that we called the residual energy: the higher the available energy, the higher is the need to “consume” this surplus in an exploratory (hence, curious) behavior. More details will be provided below and in section 4 where we present some concrete instances of the reward functions used to capture this model of curiosity.

### Action space

Analogously to Di Nocera et al. (2012), in our model, for each behavior *b* we introduce an *Action Space A*^*b*^ representing the set of possible periods *P^b^* = {*p*^*b*^_1_, …, *p*^*b*^_*k*_} for that behavior. That is, an action *a*^*b*^ is a possible assignment of a clock period *p*^*b*^ which regulates the sampling rate and the activation frequency of the associated behavior. As explained above, the idea is that the system does not learn directly the action to execute, instead, it learns the attentional policies (i.e., clock regulations with respect to its perceptual and attentional state). In this context, the action selection is an indirect consequence of the attentional behaviors. In the curiosity-driven setting, different attentional shifting strategies will be learned depending on the level of curiosity of the agent.

### State space

In order to represent the curiosity state into the state space, we reformulate the *State Space S*^*b*^ of Di Nocera et al. (2012) introducing a new parameter representing the degree of curiosity of the agent. In the extended framework, the state *s*^*b*^ is determined by a triple (*c*^*b*^, *p*^*b*^, σ^*b*^), where, *c*^*b*^ represents the level of curiosity of the system, *p*^*b*^ is for the current clock period, and σ^*b*^ is the current perceptive state of a behavior *b*. In particular, for each behavior, the attentional monitoring period *p*^*b*^ ranges in a predefined set of possible values *P*^*b*^. Analogously, the perceptive state σ^*b*^ is suitably discretized in intervals representing sub-ranges of the input signal *X*^*b*^. Finally, the curiosity degree *c*^*b*^ ranges in an interval of the four values [LL, LH, HL, HH] representing four relations between *wanting* and *liking* values (low-low, low-high, high-low, high-high) which are inspired by the curiosity model definition introduced in Litman (2005) (see section 2.2). Therefore, the attentional allocation policy π^*b*^ : *S*^*b*^ → *A*^*b*^ represents a mapping between the current state *s*^*b*^ and the next action *a*^*b*^ corresponding to the suitable period for the attentional monitoring *p*^*b*^, that is learned by means of the QL algorithm.

### Reward function

Given the Q-Learning Actions and States Spaces, we can introduce the *Reward function* as a combination of *extrinsic* component, an *intrinsic* component and a dynamic weight between these two. While the extrinsic reward depends on the direct effect of the actions with respect to the behavior utility, in our curiosity model, the second reward is directly related to the pleasure of the novelty, hence to the level of liking. Instead, the wanting level is used to dynamically balance the relation between extrinsic and liking reward: the higher the need of information seeking, the higher the liking associated with the encountered novelty. As stated before, differently from Litman (2005), our assumption is that the level of wanting depends on a sort of (global) energy state of the agent (see section 4 for additional details in the case study). The idea is that the robotic agent can explore new situations, guided by curiosity, only when the system is in a wellness state. Instead, when the system is under a certain wellness threshold, the attention is focused on priority needs (e.g., to eat and drink) rather than on secondary ones (information seeking and exploration of new states). We formalize the overall reward function as follows:

(4) $$R^{b}=(1-w)\cdot R_{e}^{b}+(w)\cdot R_{l}^{b}$$

where *R^b^_e_* is the reward computed considering the observed state, and *R^b^_l_* represents the reward evaluated considering the satisfaction of an observation with respect to a particular curiosity state (i.e., the reward is related to something that the agent likes just because it is novel). The value of *R^b^_l_* is thus computed as level of *liking*. The *w* value represents the level of *wanting*, an internal unmotivated need to explore something (the drive toward a specific location/object depends on the liking mechanism).

This relation between liking and extrinsic rewards implies that, when the situation is critical (i.e., low energy) the *R_l_* reward value will be neglected with respect to the *R_e_* extrinsic reward value, while *R_l_* will be emphasized as much as the agent will be in a wellness state. The possible correspondences between the *R_l_*, *R_e_* rewards and the *wanting*, *liking* values are illustrated in the Table 3. Notice that this matrix of wanting and liking relations is different from the one by Litman (2005), because of the different interpretation of the wanting system. For example, here low wanting and liking levels are associated with the prevalence of extrinsic rewards, while in Litman (2005) they are directly associated to a boredom state.

**Table 3:** **Wanting and liking relations and the associations between liking and extrinsic rewards**.

**Liking**	**Wanting**	
	**Low**	**High**
Low	LL: *R*_*e*_ >> *R*_*l*_	LH: *R*_*e*_ < *R*_*l*_
High	HL: *R*_*e*_ > *R*_*l*_	HH: *R*_*e*_ << *R*_*l*_

## Case study

**Figure 3:**
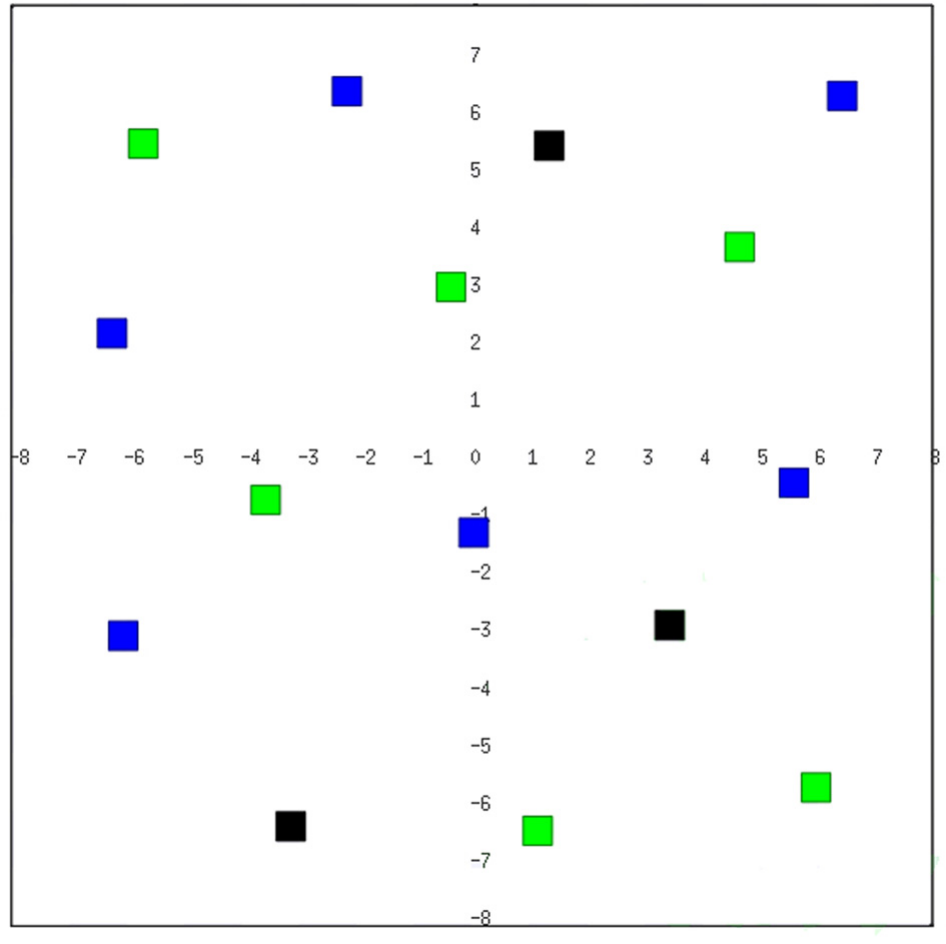
**The testing environment is simulated through the Player/Stage tool for robotics development (Gerkey et al., 2003)**. We adopt a simulated *Pioneer3-DX* mobile robot endowed with a blob camera and 16 sonar sensors. The black, blue, and green colored cubes (of size 0.5 m×0.5 m×0.5 m) within the environment represent respectively obstacles, water, and food.

In order to test our approach we introduce a *Survival Domain*, where a robot must survive for a predefined amount of time within an environment (see Figure 3) avoiding obstacles (objects, walls, etc.) and recharging energy by eating and drinking.

We consider simulated environments of 16 m^2^. Obstacles, water, and food locations are cubes of size 0.5 m×0.5 m×0.5 m, respectively of black, blue, and green color (see Figure 3). An experiment ends in a positive way if the robot is able to survive till the end of the test (*max_time*), while it fails in the following three cases: (1) the robot collides with an obstacle or its distance from an obstacle is under a certain *safety* distance threshold; (2) the value representing the robot *thirst* goes under the minimum value; (3) the value representing the *hunger* goes under the minimum value. We tested our approach using a simulated *Pioneer3-DX* mobile robot (using the Player/Stage tool Gerkey et al., 2003), endowed with a blob camera and 16 sonar sensors.

### Internal needs functions

We assume that the robot is endowed with internal drives. In our case study, we consider two internal needs: hunger and thirst. These are modeled by the following functions.

We introduce a *Hunger function*, to compute the need for food:

(5) $$\begin{array}{l} Hunger(t)=Hunger(t-1)+k\cdot (nb\_act)\\ \;-(e_{f}\cdot food\_consumed) \end{array}$$

Here, the hunger increases the need for food at each machine cycle by a *k* value, for each active behavior (*nb_act*), and decreases it when a quantity of food is ingested (*food_consumed*), depending on the energy power of the food (*e_f_*).

An analogous *Thirst function* is used to compute the need for water:

(6) $$\begin{array}{l} Thirst(t)=Thirst(t-1)+k\cdot (nb\_act)\\ \;-(e_{w}\cdot water\_consumed) \end{array}$$

#### Attentional behavior-based architecture

**Figure 4:**
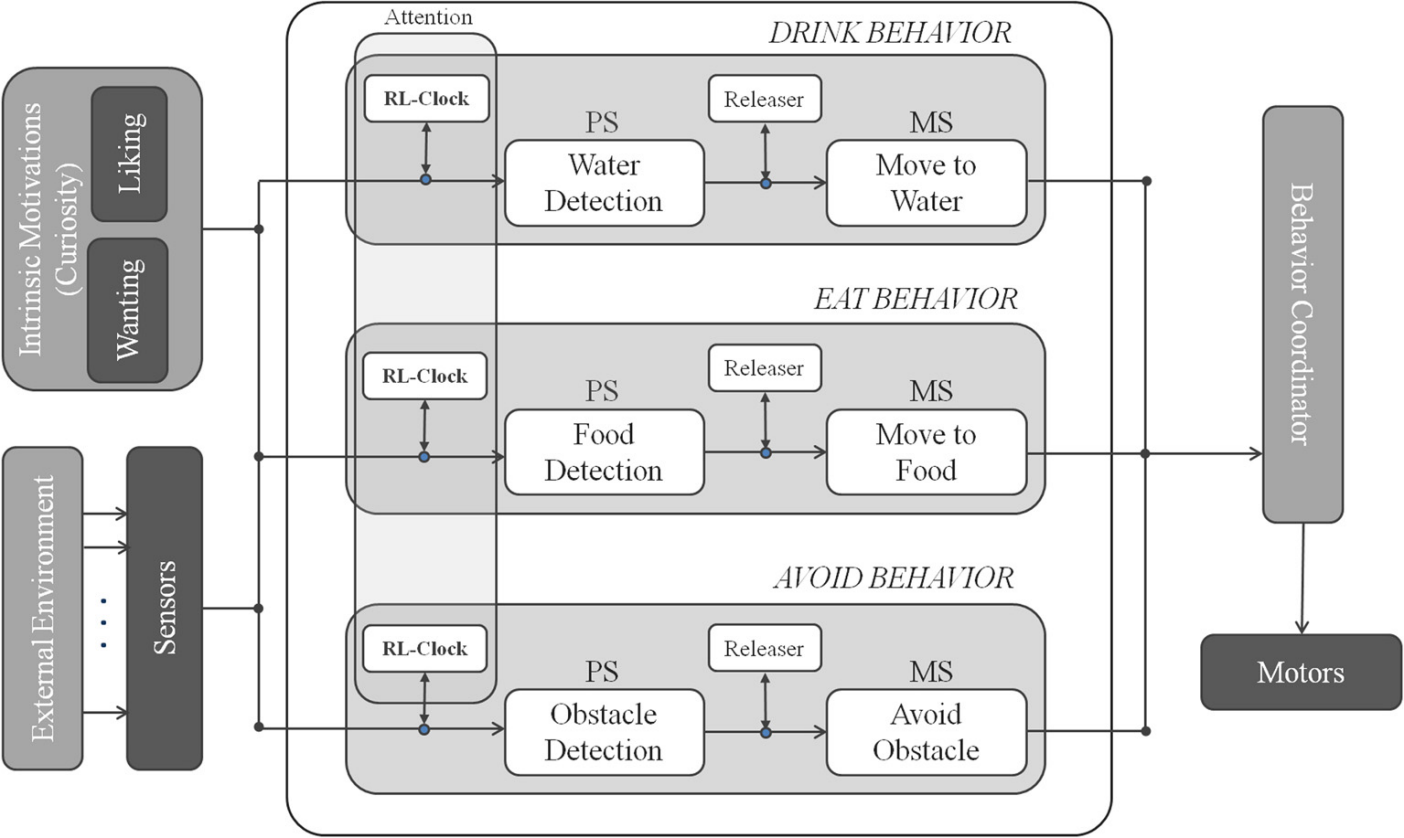
**Attentional behavior-based architecture with intrinsic motivations**.

We introduce a Behavior-Based Attentional Architecture (see Figure 4) where, the attentional control is obtained from the interaction of a set of three parallel attentional behaviors AVOID, EAT and DRINK.

For each behavior, the process of changing the rate of sensory readings is interpreted as an increase or decrease of selective attention toward internal or external saliences. These sources of salience are generally behavior- and task-dependent; these can depend on either internal states, such as hunger, thirst, etc., or external stimuli, such as obstacles, unexpected variations of the environment, attractiveness of a particular object, etc. The overall attentional behavior should emerge from the interrelation of the attentional mechanisms associated with the different primitive behaviors and learned by means of the motivated RL learning technique.

In Figure 4 we illustrate the attentional control system designed for the survival domain. It combines three behaviors: AVOID, EAT, and DRINK, each endowed with its releaser and adaptive clock. The output of the robotic system is the combination of the outputs (if they are available).

#### AVOID

Manages obstacle avoidance. Its input signal σ^*avoid*^_*t*_ is the minimum distance of the 8 frontal sonar sensors; its motor schema controls the robot linear and angular velocity (*v*(*t*), θ(*t*)) generating a movement away from the obstacle. The obstacle avoidance is obtained as follows: *v*(*t*) is proportional to the obstacle proximity, i.e., $v(t)=v_{max}\;\cdot \;\frac{\sigma_{t}^{avoid}}{\sigma_{max}^{avoid}}$, where *v*_max_ and σ^*avoid*^_max_, are respectively the maximum velocity and the maximum sonar range; θ(*t*) is obtained as weighted sum of the angular velocities generated by the active sonars, i.e., $\theta (t)=\sum_{i\;\in \;A(t)}\theta_{max}\;\cdot \;\theta_{i}$, where *A*(*t*) is the set of active sonars detecting an obstacle at time *t*, θ_max_ is the maximal rotation, θ_*i*_ is a suitable weight depending on the sonar position (high values for frontal sonars and low for lateral ones).

#### EAT

Monitors an internal function *Hunger*(*t*) representing the need of food. At each execution cycle the Hunger function changes as described in the previous section. Therefore, EAT is active when σ*^eat^_t_* = *Hunger*(*t*) goes above a suitable threshold σ*^eat^_max_*. When enabled, if a green blob (representing the food source) is detected by the camera, the motor schema generates a movement toward it, otherwise it starts looking around for the green, generating a random direction.

#### DRINK

Monitors a function *Thirst*(*t*) that represents the need of water and considers the height (pixels in the field of view) of a detected blue object in the environment as an indirect measure of the distance from the object. The motor schema is enabled whenever the σ*^drink^_t_*= *Thirst*(*t*) is greater then a suitable threshold σ*^drink^_max_*. When enabled, if a blue blob is detected by the camera, the motor schema generates a movement toward it, otherwise it starts looking around as for the EAT behavior.

For each behavior, the clock regulation depends on the monitoring function that should be learned at run-time.

### Motivated attentional framework

#### Action and state spaces

In order to cast the RL problem in our case study, we have to define *A^b^*s and *S^b^*s. In our attentional allocation problem, for each behavior, the action space *A*^b^ is represented by a set of possible periods {*p^b^_1_*, …, *p^b^_k_*} for the adaptive clock of each behavior *b*. In the case study, for each behavior (AVOID, EAT and DRINK) we assume 1 machine cycle as the minimum clock period and the following set of possible periods: *p^a^*, *p^e^*, *p^d^* = {1, 4, 8, 12}. As for the state space *S*^b^, we recall that each state is a triple (σ^*b*^, *p^b^_i_, c^b^*) composed of a value in the perceptual domain, a period, and a curiosity value. The perceptive state of each behavior is obtained as a discretization in six equidimensional intervals of the perceptive domain [σ*^b^_min_*,σ*^b^_max_*]. The perceptive domain for AVOID spans the interval [0,σ*^avoid^_max_*], where σ*^avoid^_max_* is maximum sonar range for the behavior; the domain of DRINK is [0, σ*^drink^_max_*], where σ*^drink^_max_* represents the maximum value for the Thirst function; the *EAT* domain is in [0, σ*^eat^_max_*], where σ*^eat^_max_* is the maximum state of hunger the robotic system can assume. The curiosity value ranges in the conceptual interval [LL, LH, HL, HH], where the combination of the *wanting* and *liking* parameters is considered.

#### Rewards

We assume the reward always positive except for a strong *penalty* if the system cannot survive. For the other cases the reward is computed as follows. For each behavior, the extrinsic reward has two additive components. The first evaluates the impact of frequent activations of a specific behavior. The higher is the frequency, the smaller is the obtained reward. This component is equal to zero if *p*^b^_*t*_ = *p^b^_min_*. The second component depends on the specific behavior.

In particular, concerning AVOID, each activation is rewarded directly proportional to the distance from the obstacle.

(7) $$R_{e}^{avoid}(t)=\{\begin{array}{ll} \frac{1}{2}\cdot \left[\left(\frac{p_{t}^{avoid}-p_{min}^{avoid}}{p_{max}^{avoid}-p_{min}^{avoid}}\right)+\left(\frac{\sigma_{t}^{avoid}-\sigma_{min}^{avoid}}{\sigma_{max}^{avoid}-\sigma_{min}^{avoid}}\right)\right], & \text{if !crash}\\ penalty, & \text{otherwise} \end{array}$$

As for EAT behavior, for each activation the reward is inversely proportional to the current hunger value. That is, a system that is more hungry takes a smaller reward.

(8) $$R_{e}^{eat}(t)=\{\begin{array}{ll} \frac{1}{2}\cdot \left[\left(\frac{p_{t}^{eat}-p_{min}^{eat}}{p_{max}^{eat}-p_{min}^{eat}}\right)+\left(1-\frac{\sigma_{t}^{eat}}{\sigma_{max}^{eat}}\right)\right], & \text{if !crash}\\ penalty, & \text{otherwise} \end{array}$$

Analogously, each activation of DRINK is rewarded in inverse proportion to the current value of thirst:

(9) $$R_{e}^{drink}(t)=\{\begin{array}{ll} \frac{1}{2}\cdot \left[\left(\frac{p_{t}^{drink}-p_{min}^{drink}}{p_{max}^{drink}-p_{min}^{drink}}\right)+\left(1-\frac{\sigma_{t}^{drink}}{\sigma_{max}^{drink}}\right)\right], & \text{if !crash}\\ penalty, & \text{otherwise} \end{array}$$

Following the description of section 3, we subdivide curiosity into two components dealing respectively with the feeling of *wanting* and *liking*. We associate the first one to the concept of *residual energy* for the robot body, while the second one to the level of *novelty* in the exploration of the learning states.

In particular, we assume that the *Energy* of the system is defined as follows:

(10) $$\begin{array}{l} E(t)=E(t-1)-e_{u}-e_{nb}\cdot (nb\_act)+e_{f}\cdot (food\_consumed)\\ \;+e_{w}\cdot (water\_consumed) \end{array}$$

where the current value of the energy *E*(*t*) is computed starting from the previous level of energy *E*(t−1), decremented of one unit of energy *e_u_*, which represents the energy consumed at each machine cycle. Then, we also consider the energy spent to activate each behavior *e*_nb_, where *nb_act* is the number of currently active behaviors. On the other hand, we assume increments of the energy in correspondence of consummatory behaviors such as EAT or DRINK, where the added quantity *e_f_* (*e_w_*) of energy depends on the consumed food or water (this is added when boolean conditions related to *food_consumed* consumed and *water_consumed* consumed becomes true).

**Figure 5:**
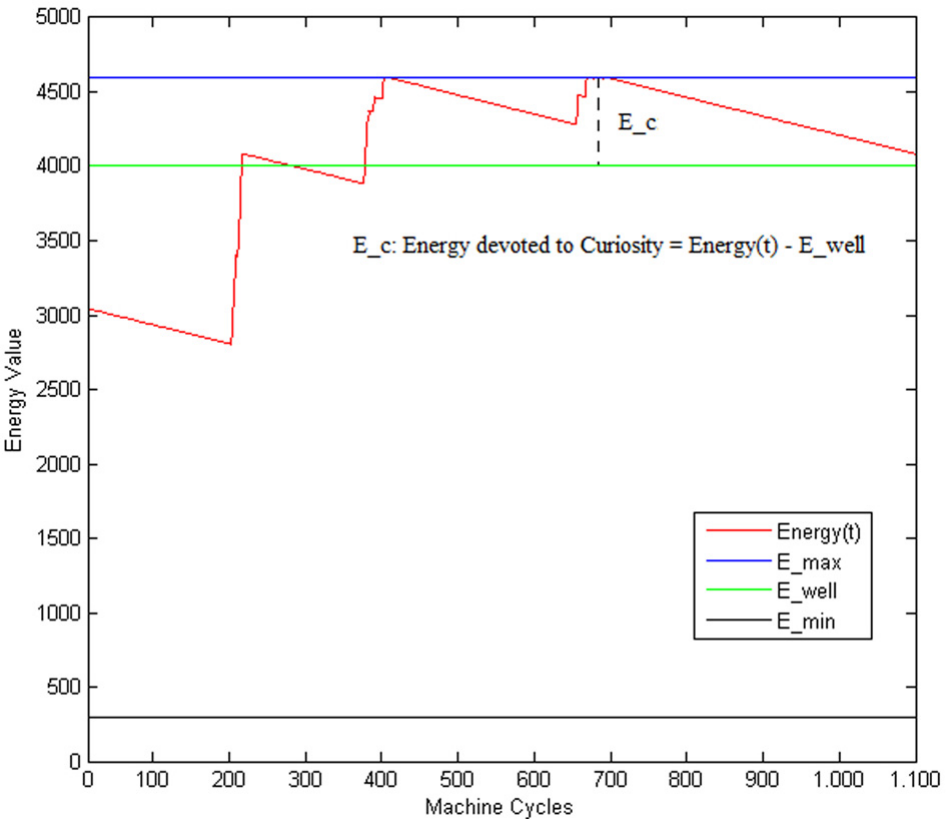
***E*(*t*) is the current Energy level; *E*_*min*: is the minimum amount of Energy permitting the system to work; *E*_*well*: is the level of Energy corresponding to a wellness state of the system**.

According to the model of curiosity considered in this paper, we model the level of the *wanting* component of the curiosity as the residue of the Energy value (see Figure 5) ranging within the interval [0,1].

(11) $$w=\{\begin{array}{ll} \frac{E(t)-E\_well}{E\_max-E\_well}, & \text{if }E(t)\geq E\_well\\ 0, & \text{otherwise} \end{array}$$

That is, the robot can show a curious behavior only when the situation is not critic (i.e., only when the global energy exceeds the *E*_well threshold, indicating a sort of *wellness* state of the system). *E*_well is supposed to be associated with a state of the system where the regulation of the different behaviors activation periods is well balanced and leads to a suitable scheduling of the actions (reach food and water when necessary while avoiding obstacles). We can interpret this residual value *E_c_* as the Energy that the system can spend on activities which are not associated with primary needs. In this way, the higher the *E_c_*, the more the curiosity can drive the system to explore new states, the less the attention is posed on the primary behaviors (such as EAT, DRINK or AVOID). According to Equations (11, 4), *c^b^* ranges only within an interval of three values [*LL*−*HL*, *LH*, *HH*]. *LL* and *HL* (i.e., both with low *wanting*) are considered as equivalent and correspond to the case of *w* equal to 0 (e.g., no curiosity).

The second component of the curiosity is the *liking*, which we associate with the pleasure due to novel situations. In particular, the curiosity in our system is interpreted as the exploration within the learning states space. We can assume that the novelty of a state is computed as follows:

(12) $$R_{l}^{b}=1-\frac{NV(\sigma_{t}^{b})}{NV\_tot}$$

where, *NV* is for number of visits and $\frac{NV(\sigma_{t}^{b})}{NV\_tot}$ represents the number of times the percept σ^*b*^_*t*_ has been observed during the previous *NV_tot_* observations. We, thus, maintain a sort of temporal window of value *NV_tot_*. In this way, on the one hand, we capture the novelty of the observation; on the other hand, we simulate a sort of lapsing mechanism where the novelty of a state is reduced when it is frequently visited within the time window. The model of the temporal window can be compared to the Itti's model of *surprise* (Baldi and Itti, 2010), by interpreting the temporal window as a rough approximation of a statistic on the perceptual history. That is, if the system is not observing a percept for *NV_tot_* times, the stimulus becomes likable again. While, if the system observes that particular perceptual state σ^*b*^_*t*_ many times (i.e., *NV_tot_*), the stimulus associated becomes boring. The *R_l_* values range in the interval [0,1], so values grater than 0.5 indicates states with high *liking*.

The combination of *wanting* and *liking* drives model the curiosity which will affect the learning system explorative attitude.

#### Parameters and settings

In Table 4 we summarize the parameters and the settings used for our experiments.

**Table 4:** **Table of the parameters experimental setting (*UoE*, Unit of Energy; *UoR*, Units of Reward; *mc*, machine cycles; *m*, meters; *s*, seconds; *obs*, observations)**.

**Experimental settings**					
**Perceptions**		**Curiosity**		**Episode**	
σ*^avoid^_max_*	1.0 *m*	*E_max*	6000 UoE (4*max_cycles)	*max_time*	180 *s*
σ*^avoid^_min_*	0.4 *m*	*E_min*	300 *UoE*	*max_cycles*	1500 mc
σ*^eat^_max_*	1500 *UoE*	*E_well*	(2/3)^*^E_max	*penalty*	−1500 *UoR*
σ*^eat^*	300 *UoE*	*NV_tot*	10 *obs*	**Power of Food/Water**	
σ*^drink^_max_*	1500 *UoE*	**Learning parameters**		*e_f_*	150 *UoE*
σ*^drink^_min_*	300 *UoE*	α	0.8	*e_w_*	150 *UoE*
**PeriodsActions**		γ	0.9	*e_u_*	1 *UoE*
*p^a^, p^e^, p^d^*	{1 mc, 4 mc, 8 mc, 12 mc}	τ	1	*e_nb_*	1 *UoE*

Here, the perceptual domain (Perceptions) and the possible periods (PeriodsActions) are analogous to the ones presented in Di Nocera et al. (2012). Indeed, this partition for the perceptive domain and the periods have been selected to obtain a satisfactory setting for the non-curious system. As for curiosity, it is associated with the residual Energy with respect to a threshold set as the 2/3 of the maximum energy *E*_*max*. The maximum energy value *E*_*max* is set with respect to the *max_cycles_* that estimates the maximum clock cycles associated with an episode (180 s to accomplish the survival task). This regulation is a compromise between scarce energy (that would keep the system in the non-curious state) and abundant energy (that would keep the system in the curious state). The minimal energy *E_min_* is set to 300. Here, for each behavior activation we have an energy consumption of 1 *UoE* while the recharge is 150 *UoE* for food and water. Concerning the liking, we employed a temporal windows of 10 observations to assess the novelty of a perceptive data. As far as the learning parameters are concerned, we set α = 0.8 for the learning rate, γ = 0.9 for the discount factor and τ = 1 for the temperature. These regulations have been defined after a preliminary phase of experimental testing in the non-curious setting (analogous to the one presented in Di Nocera et al., 2012).

## Results

In this section, we present the experimental results of a robot that must survive for a predefined amount of time within an environment (see Figure 3) avoiding obstacles (objects, walls, etc.) and meeting its energy needs by eating and drinking. We discuss the approach by considering the performance of the intrinsically motivated RL in learning attentional allocation policies in this survival domain. In particular, our aim is to evaluate the effects of the curiosity on the RL process by comparing the behavior of the curious system (from now on called CR = *Curious Robot*) with respect the one of system that is not endowed with the curiosity drive (from now on called NCR = *Non−Curious Robot*). Namely, the difference between the CR and NCR models is that the latter does not consider the rewards due to the curiosity. Notice that the parameter regulation process described in the previous section was carried out in order to obtain the best regulation for the non-curious system. Since these settings are shared by the curious and non-curious system, we can assess the added value of the intrinsically motivated framework in the testing scenario.

**Figure 6:**
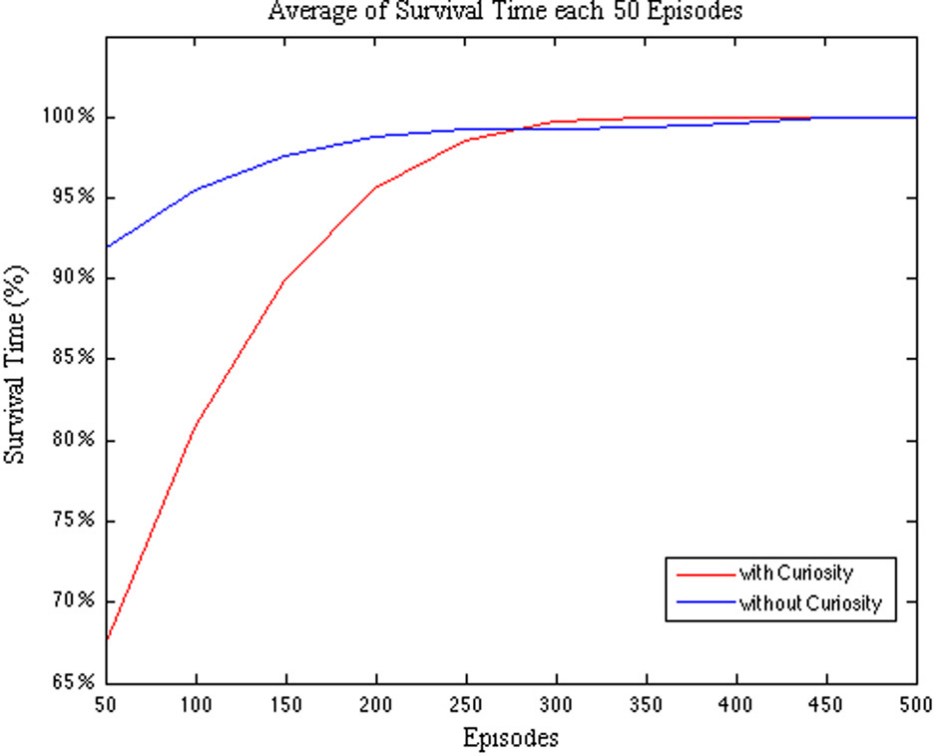
**Comparison between CR and NCR systems with respect to the *survival time* percentage per Episode**. The survival time is averaged every 50 episodes.

In order to evaluate how the curiosity affects the learning process, we first compare the survival time percentage of the CR with respect to the NCR. In Figure 6 we plot the survival time percentage averaged every 50 episodes.

As we stated before, the robot must survive in the testing environment for a predefined amount of time (*max_time*). The plot in Figure 6 shows that during the first 250 episodes the NCR system is more effective in surviving in the environment. In fact, the survival time percentage starts from a value over 90%. That is, the NCR system is more effective in action selection than the CR system. This could be due to the fact that the curiosity, initially, leads the system to prefer the exploration of novel spaces rather than the goal-directed ones. However, after a while, the CR system starts to rapidly increase its survival time until it over pass the NCR system and reaches the convergence (100% of the survival time) around the episode 300, with respect to the NCR system that does not reach the convergence before 450 episodes. Hence, in both the cases we observe that the learning converges after at most 450 episodes, however, in the case of the robot endowed with curiosity an earlier convergence is obtained.

**Figure 7:**
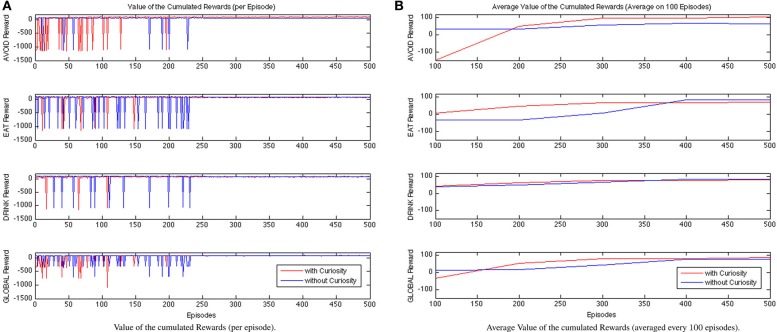
**Comparison between CR and NCR rewarding values during learning process evaluated for each episode (A) and considering the data averaged every 100 episodes (B)**.

In Figure 7, we show the cumulative rewards for each behavior during the learning process. The red lines describe the trend of the rewards gained by the system endowed with curiosity, while the blue ones are for the NCR system. As expected, during the first episodes the curious robot is not able to learn the attentional strategies needed to regulate the activations of the robot behaviors. The cumulative rewards related to the behavior AVOID of the CR system show that the performance remains unsatisfactory approximately until the episode 200. Then, the values of the cumulative rewards starts to increase and to converge from, approximately, the episode 300. In contrast, the NCR system shows a worst trend of the cumulative rewards for the EAT and DRINK behaviors. This could be explained by the fact that the robot is not guided by the curiosity to immediately explore the spaces of the environment where food or water are not observed. It only learns to eat or drink when the associated need functions (hunger and thirst) exceed a certain threshold; while the associated behaviors remains always relaxed. That is, the learned policy for the NRC DRINK behavior always selects the maximum value for the period (*p*^drink^ = 12) for all the states associated to low levels of thirst (i.e., from σ_1_ to σ_4_). On the contrary, it selects the shorter period value (*p*^drink^ = 1) for the states with a high level of thirst (σ_5_ and σ_6_). In the case of the CR robot, the process of learning is affected by the curiosity, which influences the robot behavior to explore spaces of the environment with food or water sources since it is immediately attracted by novel stimuli (including green and blue blob). Hence, the CR system learns to eat or drink also when this is not strictly required. For example, the learned policy for the CR DRINK behavior, in the case of low curiosity, associates the maximum value for *p*^drink^ only to fewer states with low levels of thirst (from σ_1_ to σ_3_), and it selects short period values (*p*^drink^ = 1) for all the other states (from σ_4_ to σ_6_). Finally, the learned policy for the CR DRINK behavior, in the case of high curiosity, always associates *p*^drink^ = 1 or *p*^drink^ = 2 to all the levels of thirst (from σ_1_ to σ_6_). At the end of the experiments NCR EAT and DRINK rewards converge to higher values, however, the global reward is higher for the CR. The global cumulative rewards are collected in the last plot of Figure 7 (on the bottom row), which shows the faster convergence of the CR system with respect to the NCR one. Finally, the CR learned policies for EAT and DRINK can always maintain the Energy value above the wellness threshold (this is also visible in Figure 10).

**Figure 8:**
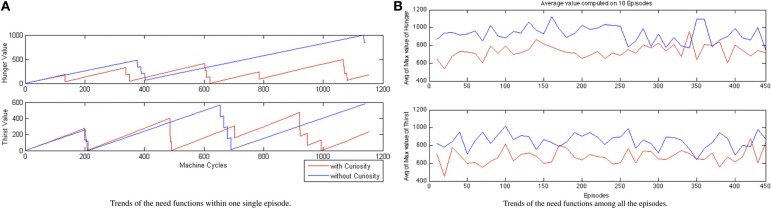
**CR and NCR need functions comparison (A) within one run (single episode) and (B) average maximum value of the Hunger/Thirst function considering the data mediated every 10 episodes**.

In Figure 8A, we show, respectively, **(A)** the trends of the need functions within a single episode after the convergence of the learning process, and **(B)** the trend of the average value of the maximum value of the need functions among all the episodes. If we look at their trends, in Figure 8A we observe some periodical path for the values of each function. We interpret the plots, in the case of the CR, as an effective learned attentional shifting policy of the behaviors EAT and DRINK. The robot seems to find a rhythmic alternation of its needs of eating and drinking (the decreasing part of the hunger and thirst functions corresponds to the consuming of food or water, respectively). On the contrary, the NCR system just waits to become very hungry or very thirsty before starting to search for sources of food and water. The behavior of the NCR robot, while on the one hand, driven by the thirst and the hunger need functions, achieves better results in terms of the single rewards, on the other hand, this does not lead to a global better reward for the NCR with respect to the CR (see Figure 7). This is also visible in Figure 8B where we can observe that the need to eat or drink for the NCR is, on average, always greater than the CR needs. Thus, the CR system is able to find a configuration of the activation periods (i.e., suitable attentional monitoring strategies), associated with the EAT and DRINK behaviors, such that the robot never suffers because of some internal need, leading to a best homeostatic regulation of the internal variables.

**Figure 9:**
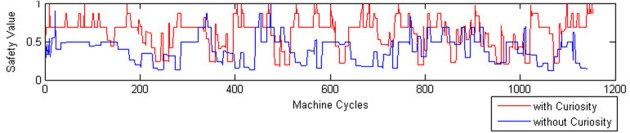
**Safety function comparison**.

**Figure 10:**
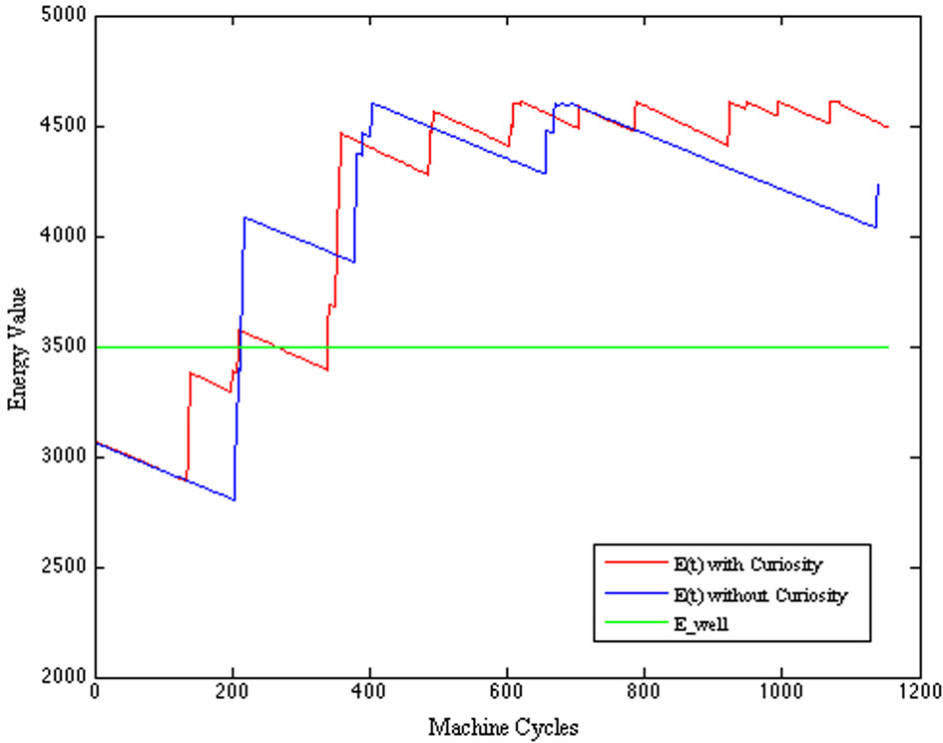
**Energy function comparison**.

In order to evaluate the performance of the two robots we defines a measure of safety as follows:

(13) $$Safety(t)=\frac{\sigma_{t}^{avoid}}{\sigma_{max}^{avoid}}\cdot \frac{p_{max}^{avoid}-p_{t}^{avoid}}{p_{max}^{avoid}-p_{min}^{avoid}}$$

where the level of safety is calculated with respect to the minimum distance between the current position of the robot and an obstacle. Here, the danger increases when the distance decreases and the AVOID activation period is relaxed; and, viceversa, the safety increases when the activation period of the AVOID is suitably balanced with respect to the distance from an obstacle. The improved performance of the CR system is visible in the evaluation of the safety function (see Figure 9), where we observe more pleasurable values for the CR robot, and of the energy function (see Figure 10), where the CR system is able to maintain the levels of energy *E_c_*with Curiosity not only above the threshold of wellness *E*_*well*, but also stabilized at a high value. The eighth row of Table 5 shows the average rewards of AVOID, EAT, and DRINK behaviors and the averages values of the global reward for the 100 episodes of validation.

**Table 5:** **Maximum, minimum and average values for the need functions (safety, hunger and thirst) and the energy function**.

	**Robot without Curiosity**				**Robot with Curiosity**			
	**Max**		**Min**		**Max**		**Min**	
Energy	4525 ± 247		2683 ± 212		4484 ± 344		2781 ± 115	
Safety	0.97 ± 0.09		0.12 ± 0.02		1.00		0.18 ± 0.04	
	**Energy**	**Safety**	**Hunger**	**Thirst**	**Energy**	**Safety**	**Hunger**	**Thirst**
Average value	3809 ± 335	0.38 ± 0.02	420 ± 181	363 ± 135	3720 ± 312	0.61 ± 0.03	328 ± 142	311 ± 137
	**Avoid**	**Eat**	**Drink**	**Global**	**Avoid**	**Eat**	**Drink**	**Global**
Reward	66 ± 2	83 ± 6	85 ± 5	235 ± 7	100 ± 9	70 ± 7	78 ± 11	248 ± 15
Activation	109 ± 3	105 ± 7	103 ± 2	260 ± 22	204 ± 19	130 ± 10	156 ± 10	418 ± 22

Average values of the cumulative eat and drink rewards and of number of activations of the behaviors after the learning process.

All the results of the above plots are summarized in Table 5, where we evaluate the average values and the standard deviations on 100 episodes used to validate our system (after the convergence of the learning process). Regarding the global energy of the system, we already noticed that such values stabilized on a specific interval for the CR (see Figure 10). In Table 5, we can find that the average of the Energy mean values (= 3720) is a bit smaller than the NCR case (= 3809) and that its maximum value (= 4488, which is above the wellness threshold *E*_*well*) is smaller than the NCR energy maximum value (= 4525). However, we suppose that the CR average of the energy mean values is smaller because of the curiosity (residual energy), which is “consumed” for exploring new states during the learning process. Interestingly, such an exploration of new states does not imply that the robot is less cautious in moving around. Indeed, the safety average value of the CR is almost two times greater (= 0.61) than the NCR (= 0.38). Moreover, the minimum value for the CR safety (= 0.18) is higher (so, more safer) than the NCR value (= 0.12). Finally, as noted in the plots (see Figure 8A) the need functions of hunger and thirst have smaller average values for the CR (hunger = 328 and thirst = 311), which means that the robot satisfies its needs more frequently.

Both CR and NCR are effective in spending computational resources. This can be observed by considering the last row of Table 5, where we show the average number of the behavior activations. The CR has a slightly greater number of activations, in particular for the AVOID behavior. This leads to an emergent behavior consistent with what discussed above. The CR robot eats and drinks more frequently and shows a safer behavior with respect to the NCR. However, 204 activations out of *max_cycles* (around 1150 in this specific case) possible activations (machine cycles of an episode) seems a satisfactory result for a behavior-based architecture (i.e., there is a reduction of the 83% of the number of activations).

Moreover, notice that the global value shown at the end of this row states for how many cycles at least one behavior was active during the episode. In the case of NCR, this value is equal to 260. This shows that it is frequent to find more than one behavior active at the same time. For the CR robot, this value is equal to 418, meaning that for the most of the time only one behavior is active and the robot is able to orchestrate the multiple behaviors by opportunely shifting attentional resources, from time to time, toward the most salient one according to its need functions.

**Figure 11:**
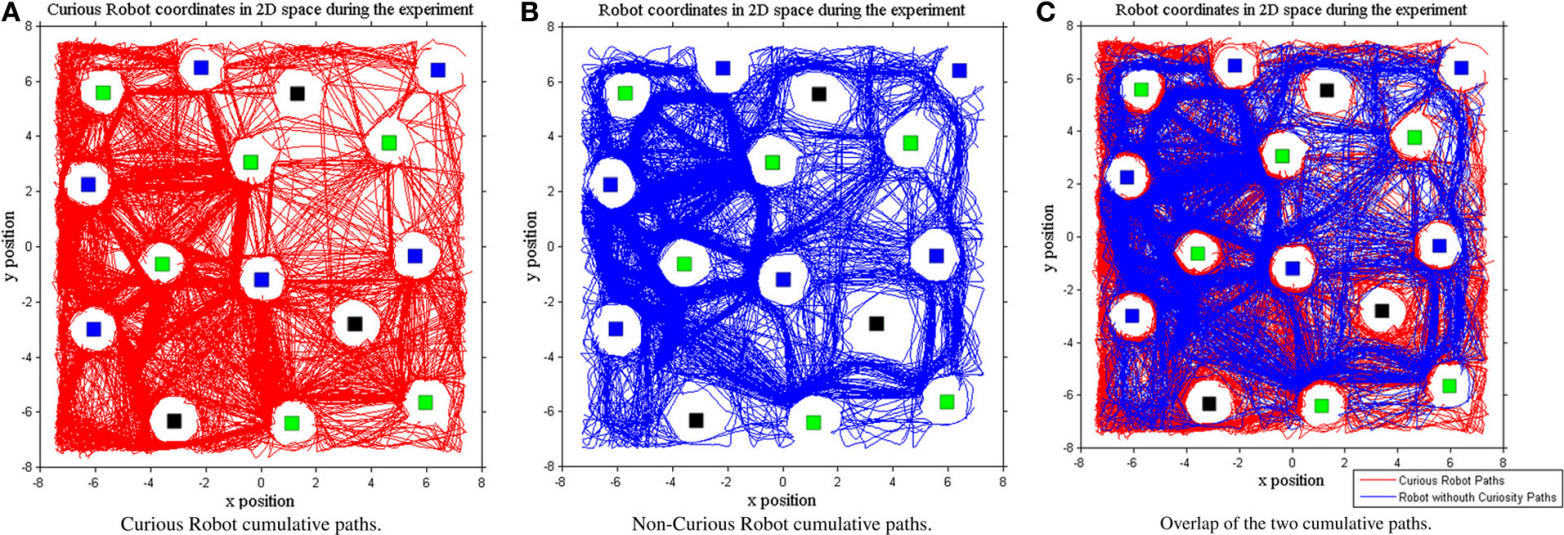
**Space of the environment explored during the Curious (A—red line) or Non-Curious (B—blue line) Learning Systems execution**. The third graph represents the comparison between the two systems **(C)**.

Finally, another interesting result regards the curiosity influence on the actual environmental exploration space. Indeed, while we expected that our intrinsic motivated RL would lead the learning process to improve the exploration of the internal learning states, we did not expect that this would also produce an increased spatial exploration of the environment. This result can be illustrated by plotting the paths of the two systems during the overall experimentation (see Figure 11C). By comparing the two generated paths, we can note that the system endowed with internal motivation (see Figure 11A) is more explorative (the cumulative traces of 500 episodes covered the 50% of the total area) with respect to the non-curious one (44% of the total area covered as shown in Figure 11B). The CR path seems smoother with a better coverage of the space around obstacles, food, and water while keeping the robot safe.

## Discussion

In this paper, we presented an intrinsically motivated RL approach to attention allocation and shifting in a robotic system. The framework has been demonstrated at work in a survival domain. Differently from classical RL models of action selection, where actions are chosen according to the operative/perceptive contexts, in our case the action selection is mediated by the attentional status of the robotic behaviors. In the literature we can find intrinsically motivated RL system where simple attentional control mechanisms are involved (e.g., eye movement in the playground domain in Barto et al., 2004); in this paper we tackle the attention allocation and shifting problem, which is novel in this context. Indeed, in our setting, the learning process is to adapt and modulate the attentional strategies used to allocate attentional resources of the system. Specifically, our attentional mechanism regulates the behavioral activation periods, hence the amount of computational and operation resources dedicated to monitor and control the associated activities. Following this approach, the global behavior of the system is not directly generated by an action selection policy (as in typical RL approaches to action selection Sutton and Barto, 1998 and intrinsically motivated RL Barto et al., 2004; Singh et al., 2004), instead, it emerges as the sum of the outputs of multiple parallel processes, each activated with its own frequency: the smaller the activation period of a behavior, the higher its influence on the global emergent behavior. Following the taxonomy proposed by Baldassarre and Mirolli (2013), our system can also be considered as a competence-based system where the skill to be learned is the attentional allocation policy, however, this policy has only an indirect effect on the overall expected reward.

As the main intrinsic motivation, we considered the curiosity drive which is inspired by the one proposed by Litman (2005). This model allows us to account for both optimal arousal and curiosity-driven approaches to curiosity modeling. In particular, we related the liking and wanting drives of the Litman's model to, respectively, the pleasure of the novelty and the residual energy of the system (the higher the energy value over the wellness state, the higher the drive toward to the exploration of novel situations and states). While several models for novelty-based and knowledge-based (Schmidhuber, 1991; Singh et al., 2004) curiosity have been proposed in the intrinsically motivated RL literature, the employment of the Litman account is less explored. Notice that we do not employ knowledge-based curiosity models (Schmidhuber, 1991; Singh et al., 2004). Indeed, while in Schmidhuber (1991) and later in Singh et al. (2004) and Oudeyer and Kaplan (2007) curiosity should lead the agent to explore areas of the environment where the learning progress is expected to be high, in our system, the agent is directly attracted by novel stimuli as sources of saliency. We want to stress here that the attentional problem addressed in our work is different from the ones mentioned since we learn attentional allocation only. In contrast to Schmidhuber (1991), Singh et al. (2004), and Oudeyer and Kaplan (2007), we can only enhance attention with respect to the attracting stimuli, but the movement of the system toward the stimuli is obtained as an indirect effect. As for the novelty, the lapsing mechanism we defined for the liking function (the novelty of a state is reduced when it is frequently visited within the time window) can be related to the Itti's model of surprise (Baldi and Itti, 2010), but also to the approach proposed by Oudeyer and Kaplan (2007), where, once predictions within a given part of the sensorimotor space are learned, the system gets bored and starts to execute other actions. As far as attentional allocation and shifting is concerned, RL models have been mainly proposed for visual attentions and gaze control (Bandera et al., 1996), a theoretical link between visual attentional exploration and novelty-based intrinsic motivations is investigated in Schlesinger (2012) where the author investigates the way in which goal directed, top–down attentional skills can be incrementally learned exploiting complex novelty detection strategies. Differently from these cases, here we investigated an intrinsically motivated RL approach to the generation of attentional strategies that are suitable for the executive control.

Our approach has been illustrated and tested in a simulated survival domain, where a robot was engaged in survival tasks such as finding food or water while avoiding dangerous situations. In this context, our goal was to show the feasibility and the effectiveness of the approach in a typical robotic domain where basic needs satisfaction and intrinsic (curiosity) motivations were clearly defined. In particular, we compared the performance of the intrinsically motivated RL with respect to the same setting except for the fact that the influence of the intrinsic drive was neglected. The parameter tuning was provided in order to find the best regulation of the non-curious setting to assess the added value of the curiosity drive. The collected results supportsthe hypothesis that the curiosity-driven learning system permits to find satisfactoryregulations of the attention allocation and shifting policies, providing different attentional policies,and consequently different emergent behaviors, depending on the current level of curiosity. Moreover, the overall behavior that emergesfrom the execution of the learned attentional policies seems safer and capable of keeping therobotic system in a higher wellness state during the environment exploration. This is related to thefact that the curiosity drive stimulates the attention toward opportunitiesof energy recharging (food and water) more frequently than in the non-curious system. Wealso observed that the curious system provides a more uniform exploration of the environment when compared with the non-curious behavior. While the presented tests illustrate the feasibility and effectiveness of the approach in a typical survival domain, the extension of this curiosity-driven attentional regulation method to more complex domains and more structured tasks (e.g., considering hierarchical skills Barto et al., 2004 and top–down attentional regulations Schlesinger, 2012) remains to be investigated as future work.

### Conflict of interest statement

The authors declare that the research was conducted in the absence of any commercial or financial relationships that could be construed as a potential conflict of interest.
